# Recovery after long‐term summer drought: Hydraulic measurements reveal legacy effects in trunks of *Picea abies* but not in *Fagus sylvatica*

**DOI:** 10.1111/plb.13444

**Published:** 2022-06-30

**Authors:** T. Knüver, A. Bär, A. Ganthaler, T. Gebhardt, T. E. E. Grams, K.‐H. Häberle, B. D. Hesse, A. Losso, I. Tomedi, S. Mayr, B. Beikircher

**Affiliations:** ^1^ Department of Botany University of Innsbruck Innsbruck Austria; ^2^ Technical University of Munich School of Life Sciences Professorship for Land Surface‐Atmosphere Interactions AG Ecophysiology of Plants Freising Germany; ^3^ Technical University of Munich School of Life Sciences Chair of Restoration Ecology Freising Germany; ^4^ Hawkesbury Institute for the Environment Western Sydney University Richmond Australia

**Keywords:** climate change, drought legacy, drought recovery, electrical resistivity tomography, forest drought, tree hydraulics, tree water storage

## Abstract

- Climate change is expected to increase the frequency and intensity of summer droughts. Sufficient drought resistance, the ability to acclimate to and/or recover after drought, is thus crucial for forest tree species. However, studies on the hydraulics of mature trees during and after drought *in natura* are scarce.
- In this study, we analysed trunk water content (electrical resistivity: ER) and further hydraulic (water potential, sap flow density, specific hydraulic conductivity, vulnerability to embolism) as well as wood anatomical traits (tree ring width, conduit diameter, conduit wall reinforcement) of drought‐stressed (artificially induced summer drought *via* throughfall‐exclusion) and unstressed *Picea abies* and *Fagus sylvatica* trees.
- In *P. abies*, ER indicated a strong reduction in trunk water content after 5 years of summer drought, corresponding to significantly lower pre‐dawn leaf water potential and xylem sap flow density. Vulnerability to embolism tended to be higher in drought‐stressed trees. In *F. sylvatica*, only small differences between drought‐stressed and control trees were observed.
- Re‐watering led to a rapid increase in water potentials and xylem sap flow of both drought‐stressed trees, and to increased growth rates in the next growing season. ER analyses revealed lower trunk water content in *P. abies* trees growing on throughfall‐exclusion plots even 1 year after re‐watering, indicating a limited capacity to restore internal water reserves. Results demonstrated that *P. abies* is more susceptible to recurrent summer drought than *F. sylvatica*, and can exhibit long‐lasting and pronounced legacy effects in trunk water reserves.

## Graphical abstract

In contrast to *Fagus sylvatica*, *Picea abies* showed drought legacy effects in hydraulics and internal water reservoirs after one‐year recovery from recurrent long‐term summer drought.

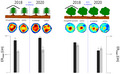

## Introduction

Forests, which are economically and ecologically valuable ecosystems, are worldwide impacted by land‐use and climate change (Allen *et al*., 2010). As a consequence of global warming, both frequency and intensity of drought events are expected to increase in Europe and many other regions (Fuhrer *et al*., 2006; Alcamo *et al*., 2007), which will have negative consequences for forest health and survival. Numerous studies have directly linked drought events to tree dieback in the last decades (Allen *et al*., 2010; Anderegg *et al*., 2013; Nardini *et al*., 2013; Adams *et al*., 2017; Cailleret *et al*., 2017; Choat *et al*., 2018) and, recently, summer droughts in 2018 and 2019 strongly impacted Central European forests (Hari *et al*., 2020; Schuldt *et al*., 2020; Salomón *et al*., 2022).

Despite the high socio‐ecological importance of forests, current knowledge on acclimation and recovery capacity of trees to drought is still limited. Numerous studies have analysed drought tolerance and recovery in juvenile, potted plants, which may not sufficiently reflect the situation in mature trees of natural stands (Magnani *et al*., 2000; Choat *et al*., 2018), *e.g*. as tree age may substantially influence stress responses (Anderegg *et al*., 2015; Nolan *et al*., 2021). Field studies on mature trees in natural stands are thus crucial to validate model frameworks developed from manipulative experiments on young plants (McDowell *et al*., 2013; McDowell *et al*., 2019; Ruehr *et al*., 2019) but remain scarce (*e.g*. Choat *et al*., 2018; Arend *et al*., 2021; Nolan *et al*., 2021). Furthermore, only a few studies have included long‐term stress, *i.e*. drought stress over several growing seasons.

In temperate regions, relevant drought events mainly occur in summer (Hari *et al*., 2020; Schuldt *et al*., 2020) when atmospheric drought and high temperatures increase the evaporative demand (Grossiord *et al*., 2020) and, in combination with low soil water content, strongly impact plant water relations. Driven by transpirational water loss over leaves, water is passively transported from the soil through the plant to the leaves (Tyree & Zimmermann, 2002). Thereby, water is in a metastable state and intact, continuous water columns are required to maintain the integrity of the water transport system. Although plants are able to strongly reduce water loss *via* stomatal closure, water can still be lost through cuticular and peridermal transpiration (Beikircher & Mayr, 2009; Beikircher & Mayr, 2013). Unless sufficiently buffered by water reserves within the plant and in the soil, tensions in xylem water columns can reach critical levels (*i.e*. xylem pressure or water potential), which lead to breakage of water columns, resulting in air‐filled conduits (*i.e*. embolism; Beikircher *et al*., 2010; Timell *et al*., 2002; Tyree & Sperry, 1989; Tyree & Zimmermann, 2002). Xylem pressure thresholds, at which embolism occurs, differ between (Charra‐Vaskou *et al*., 2012; Choat *et al*., 2012; Nolf *et al*., 2016) and within (Alder *et al*., 1996; Beikircher & Mayr, 2009; Nolf *et al*., 2016; Skelton *et al*., 2017; Lucani *et al*., 2019) species, as well as among different organs within a plant (Cochard *et al*., 1999; Beikircher & Mayr, 2008; Losso *et al*., 2019). Recent studies also show that thresholds vary with plant age (Hammond *et al*., 2019; Zhang *et al*., 2020). Embolism reduces xylem transport capacity, which may induce a vicious cycle of further decreasing xylem pressure and spreading of embolism, possibly resulting in plant death (Tyree & Sperry, 1988; Adams *et al*., 2017; Choat *et al*., 2018; Tomasella *et al*., 2018). Several studies indicate a point of no return between 50% and 80% loss of conductivity in gymnosperms and between 80% and 100% in angiosperms (Brodribb & Cochard, 2009; Brodribb *et al*., 2010; Urli *et al*., 2013; Choat *et al*., 2018; Hammond *et al*., 2019), although these thresholds are not only species‐ but also stress‐specific (*e.g*. winter stress, repeated drought; Feng *et al*., 2021; Mayr *et al*., 2019). The ability to maintain xylem pressure above critical thresholds and to maintain long‐distance water transport is thus a key component of drought tolerance (McDowell *et al*., 2008). However, closed stomata not only ensure hydraulic integrity but also reduce carbon uptake and thus can lead to carbon starvation (McDowell *et al*., 2019). This can pose a considerable risk for isohydric species that attempt avoid hydraulic failure by closing stomata even at moderate xylem pressures. Under long‐term drought this strategy might render plants more susceptible to carbon starvation (McDowell & Sevanto, 2010; Blackman *et al*., 2019). Isohydric behaviour is often reported in conifers (*e.g*. Norway spruce; Grams *et al*., 2021; Lyr *et al*., 1992; Rötzer *et al*., 2017), while many hardwoods show more anisohydric behaviour (Beikircher *et al*., 2013; Rötzer *et al*., 2017; Leuschner, 2020; Grams *et al*., 2021). Under drought, such trees close stomata late, which allows prolonged maintenance of carbon supply, but puts them at risk of hydraulic failure, especially during short‐term severe drought stress (McDowell *et al*., 2008; Anderegg *et al*., 2013; Choat, 2013; Hartmann *et al*., 2013; Sevanto *et al*., 2014; Martin‐StPaul *et al*., 2017; Anderegg *et al*., 2018; Blackman *et al*., 2019). Stomatal regulation is only one aspect of several, usually coordinated, components of plant water relations, including water uptake and internal water reserves (*e.g*. water stored in the trunk) that are important in buffering short‐ and long‐term water deficits.

Acclimation may help plants to cope with recurrent drought events by adjustments in physiological and anatomical traits (Beikircher & Mayr, 2009; Montwé *et al*., 2014). For instance, smaller conduits with increased cell wall reinforcement and pits with smaller pores and/or higher pit‐membrane thickness (Hacke *et al*., 2001; Jansen *et al*., 2012; Montwé *et al*., 2014; Gleason *et al*., 2016) can increase the hydraulic safety (*i.e*. ability to avoid embolism formation). In contrast, repeated formation and repair of embolism may also reduce hydraulic safety and therefore induce drought‐related legacy effects (Feng *et al*., 2021).

After drought, the capacity to recover is crucial for plants: recovery is defined as the difference between pre‐stress (or control conditions) and post‐stress and can be partial, complete or compensatory (Ruehr *et al*., 2019). Stress intensity and duration influence physiological impairment and/or structural damage and impact speed of recovery, which also varies among different traits (Mencuccini, 2003; Skelton *et al*., 2017; Choat *et al*., 2018; Ruehr *et al*., 2019). Severe drought stress can lead to irreversible damage to living tissues, depletion of non‐structural carbohydrates (NSC) and massive conductivity losses that are critical for recovery and can even result in plant death. Furthermore, prolonged drought periods and more frequent droughts reduce time available for recovery between stressful periods, and thus can negatively impact survival (Schwalm *et al*., 2017). Recovery of the hydraulic system also often involves the formation of new conduits or resprouting, which may be delayed to the next vegetation period (Christensen‐Dalsgaard & Tyree, 2014; Zeppel *et al*., 2015; Choat *et al*., 2018; Ruehr *et al*., 2019). However, our knowledge on the capacity of mature trees to recover after drought is still limited (Choat *et al*., 2018; Ruehr *et al*., 2019; Brodribb *et al*., 2020).

In the present study, we analysed various hydraulic and related wood anatomical traits of adult trees during and after drought. Within the framework of the ‘Kranzberg Forest Roof experiment’ project (KRoof; Grams *et al*., 2021; Pretzsch *et al*., 2014; Pretzsch *et al*., 2020), mature trees of *Picea abies* (Norway spruce) and *Fagus sylvatica* (European beech), growing in a natural Central European forest stand, were subjected to summer droughts using throughfall exclusion of precipitation. After 2 years of summer drought, Tomasella *et al*. (2018) reported significant shifts in various hydraulic parameters (pre‐dawn water potential, vulnerability thresholds). In both species, drought‐stressed trees showed reduced growth, but higher drought tolerance compared to control trees. After 5 years of summer drought, we analysed the hydraulics of the same trees again, and also monitored changes in various hydraulic and anatomical parameters during and after re‐watering. Specifically, we analysed trunk water content as a central internal water buffer, water potential and sap flow density 1 year before, during and 1 year after re‐watering. Furthermore, we determined the hydraulic safety and efficiency at maximum drought stress intensity 1 year after re‐watering and linked the findings to xylem anatomical parameters. We hypothesized that long‐term drought stress will strongly impact tree hydraulics, and that the effects will be more pronounced in Norway spruce than in European beech. We further hypothesized that both species would be able to recover, with rapid responses of physiological traits (*e.g*. trunk water storage, water potential, sap flow density) and – based on xylem anatomical changes – distinct adjustments in hydraulic efficiency and safety within 1 year after the last drought episode. Outcomes should provide insights into the acclimation potential of mature trees as well as their capacity to recover after repeated, long‐term summer droughts.

## Material and Methods

### Experimental set up and plant material

The study site, Kranzberg Forest, in southern Germany (48°25′12′′N, 11°39′42′′W, 490 m a.s.l.), consists of a mixed stand of European beech (*Fagus sylvatica* [L.]; mean age in 2020: 89 ± 4 years) and Norway spruce (*Picea abies* [L.] Karst; mean age in 2020: 69 ± 4 years; Pretzsch *et al*., 2014). Long‐time average (1971–2000) mean annual air temperature, mean annual precipitation, and mean air temperature and precipitation during the growing season (May to September) are 7.8 °C and 13.8 °C, with 750–800 mm year^−1^ and 460–500 mm year^−1^, respectively. (Grams *et al*., 2021. Within the framework of the ‘Kranzberg Forest Roof experiment’ (KRoof; Goisser *et al*., 2016; Grams *et al*., 2021; Pretzsch *et al*., 2014; Pretzsch *et al*., 2016), the study site was divided into 12, 110 to 200 m^2^ plots, each containing three to seven *F. sylvatica* and three to seven *P. abies* mature trees. In spring 2010, the plots were trenched to 1‐m soil depth, to a dense layer of tertiary sediments. The trenches were subsequently lined with plastic tarpaulin (waterproof and impermeable to root growth) and refilled with soil. From 2014 to 2019, rainfall was excluded with roofs at ~3 m above ground level (throughfall exclusion; TE) in six of the 12 plots. From April/May to November, the roofs automatically closed during precipitation, whereas in winter, roofs were permanently open (Grams *et al*., 2021). In late June/early July 2019, TE plots were re‐watered with *ca*. 12849 ± 2801 l of water. As high hydrophobicity of the topsoil layer in TE plots only allowed a slow rate of re‐watering (2 l m^−2^ h^−1^), re‐watering took about 40 h. To minimize the effect of re‐watering on soil temperature and nutrient availability between control (CO) and TE plots, the CO plots were also watered, although to a minor extent (*ca*. 2035 ± 537 l; Grams *et al*., 2021). Re‐watering was conducted in three campaigns (25 June, 4 July and 10 July 2019), in which two CO and two TE plots were re‐watered at each time. (For further details on experimental set up and re‐watering, see Grams *et al*., 2021).

**Fig. 1:**
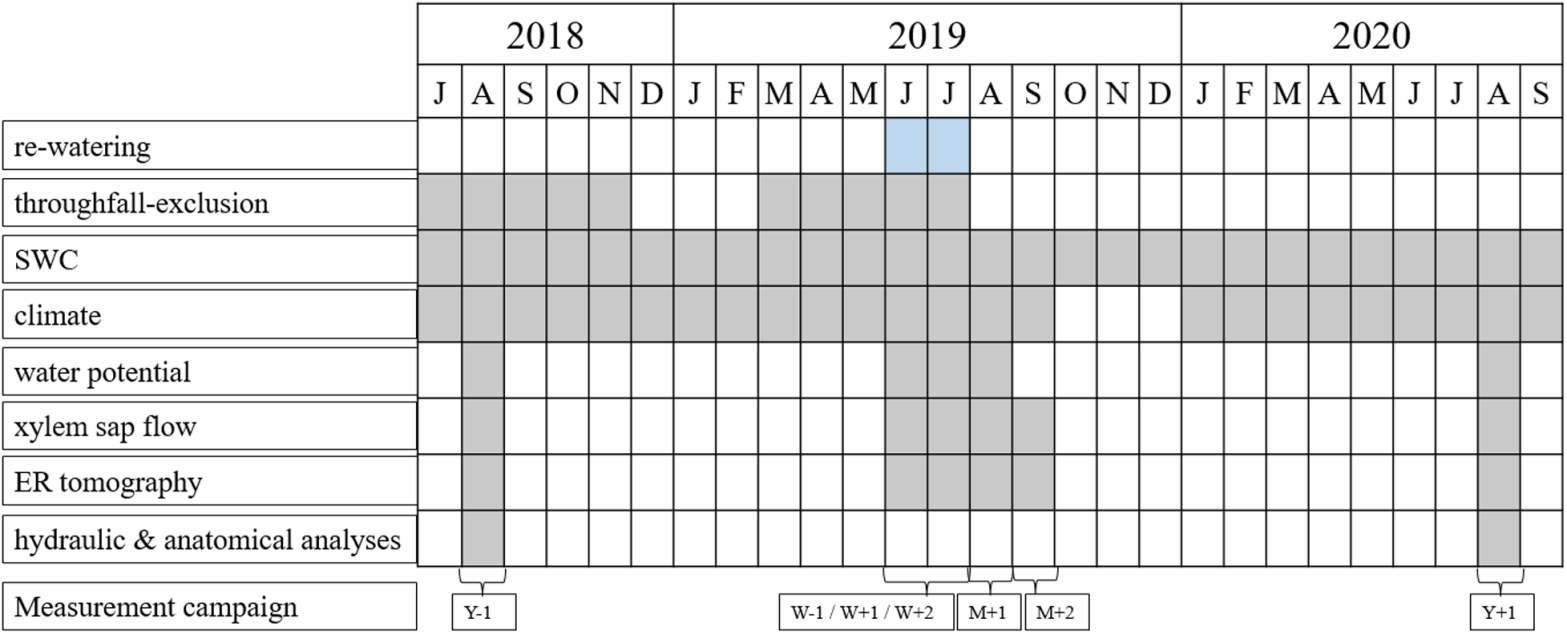
Overview of measurements carried out in the framework of the present study. Y‐1 = 1 year before and W‐1 = 1 week before re‐watering; W + 1 = 1 week, W + 2 = 2 weeks, M + 1 = 1 month, M + 2 = 2 months and Y + 1 = 1 year after re‐watering. No climate data are available for Oct–Dec 2019 due to maintenance of devices.

Before, during and after re‐watering, pre‐dawn water potential (Ψ_pd_), xylem sap flow density and trunk water content were analysed on seven specific dates: 1 year (Y‐1, *i.e*. maximum drought stress) and few days (W‐1) before re‐watering, 1 and 2 weeks (W + 1, W + 2), 1 and 2 months (M + 1, M + 2), and 1 year (Y + 1) after re‐watering (see Fig. 1). Furthermore, vulnerability to drought‐induced embolism, specific hydraulic conductivity and xylem anatomy were analysed during the maximum drought stress (August 2018; Y‐1), and 1 year after re‐watering (August 2020; Y + 1; Fig. 1).

### Climate and soil volumetric water content

From January 2018 to October 2020, precipitation, vapour pressure deficit (VPD) and air temperature were acquired from the experimental site meteorological station (Umwelt‐Geräte‐Technik, Münchberg, Germany). VPD and air temperature were measured at canopy height (Grams *et al*., 2021).

Soil volumetric water content (SWC, vol. %) was measured once per week *via* time domain reflectometry (TDR 100; Campbell Scientific, Logan, UT, USA). In the present publication, we provide data from two TDR probes installed in the middle of each plot at 10–30 cm and 30–50 cm. For data on further locations and depths, see Grams *et al*. (2021).

### Pre‐dawn water potential and xylem sap flow density

Pre‐dawn water potential (Ψ_pd_; MPa) was measured on 6–8 trees per treatment and species using a Scholander pressure chamber (model 1505D; PMS Instrument, Albany, NY, USA). End twigs were manually harvested between 03:00 and 04:00 h, solar time, from sun‐exposed crown branches at a height of ~30 m *via* a crane. Immediately after harvesting, samples were sealed in plastic bags to avoid water loss during transport from the crown to ground level, where Ψ_pd_ was measured.

Xylem sap flow density (xylem sap flow per unit sapwood) in the outer xylem (0–20 mm) was measured at breast height using the heat dissipation method (Granier, 1985; Granier, 1987). On each of the electrical resistivity tomography measurement trees, two sensors were installed (south and north exposure) and data were averaged to account for differences within each tree in order to calculate the xylem sap flow density (l dm^−2^ day^−1^) per tree. Rainy days (>10 mm precipitation day^−1^) were excluded from the calculation.

### Electrical resistivity tomography

To monitor trunk water content before, during and after re‐watering *in situ*, 6 to 8 trees per treatment and species, growing in at least four different plots per treatment, were analysed *via* electrical resistivity (ER) tomography. Following the protocol given in Bär *et al*. (2019), 24 stainless steel nail probes were installed around the circumference of a given tree at breast height (~130 cm). The nails (sterilized before use) were inserted into the trunk until contact with the sapwood was established. The position of the nails, and thus the geometry of tree trunks, was determined with an electronic calliper (PiCUS Calliper Standard Version; Argus Electronics, Rostock, Germany) and processed with the PiCUS software (PiCUS Q73, Argus Electronics). *Via* electrodes, the nails were connected to a 24‐channel resistivity system (PiCUS TreeTronic; Argus, Electronic) and voltage applied. Voltage levels were set to 8 for *P. abies* and 4 for *F. sylvatica*. After measurement of the electrical field in the trunk, data were processed with the PICUS software to calculate the distribution of ER in the trunk cross‐section, and to generate the respective tomogram. Tomograms consist of triangles, coloured according to their resistivity values. The underlying spatial distribution of resistivities is based on an inversion scheme that uses a finite element simulation operating with regularly arranged tetrahedrons (Günther *et al*., 2006; Rücker *et al*., 2006). The source data are processed into a 2D model by the software and provide a planar triangular‐based mesh at the measurement level. Triangle size, position and respective ER value were exported for further analysis. As each triangle area varies in dependence on its radial position, the weighted ER (ER_W_; Ωm) was calculated for each triangle:

(1) $${\mathrm{ER}}_{W}=\left(\mathrm{ER}\;x\;A\right)/A_{\text{mean}}$$

where A (cm^2^) is the individual triangle area and A_mean_ (cm^2^) is the mean area of all triangles. Based on the ER_w_ values of single triangles, the average ER of the entire cross‐section (ER_mean_) was then calculated. The outer 5% of the trunk geometry was removed from the calculation to exclude artefacts related to nail insertion.

### Xylem hydraulic efficiency and safety

In August 2018 and August 2020 (*i.e*. 1 year before and 1 year after re‐watering), maximum specific hydraulic conductivity and vulnerability to drought‐induced embolism were analysed on five trees per species and treatment, growing in at least two different plots per treatment. Harvesting and sample preparation were done following Beikircher & Mayr (2016). Briefly, about 80‐cm (*F. sylvatica*) and 60‐cm (*P. abies*) long, sun‐exposed branches were collected at ~30‐m height using a canopy crane, immediately placed in water‐filled buckets, covered with a dark plastic bag and re‐cut twice under water at about 8 cm (*F. sylvatica*) and 2 cm (*P. abies*). For transport (within 1 day) to the laboratory at the University of Innsbruck, branches were tightly wrapped in dark plastic bags containing wet paper towels. In the laboratory, leaves and side twigs were removed under water for vulnerability and conductivity analyses. The apical parts of each branch (*P. abies*: 3.4 ± 0.3 cm; *F. sylvatica*: 27.4 ± 1.9 cm) were used for conductivity analyses, and the subsequent 30 cm were used for vulnerability analyses.

To measure maximum specific hydraulic conductivity (*k*_S_; cm^2^ s^−1^ MPa^−1^), the basal end of each sample, cut from current‐year shoots of harvested branches (see above), was debarked (about 1 cm) and both sample ends trimmed with a sharp woodcarving knife. Then, the basal end was tightly sealed in a tube connected to a Coriolis mass flow meter (mini CORI‐FLOW M13 100 g h^−1^; Bronkhorst Cori‐Tech, Ruurlo, Netherlands). After measuring the native hydraulic conductance at a pressure of 0.006 MPa, samples were repeatedly flushed at 0.8 MPa (*P. abies*) and 1.0 MPa (*F. sylvatica*) for 30 min (to remove native embolism) until stable conductance values were reached. Measurement solution consisted of distilled, filtered (0.22 μm) and degassed water containing 0.005% (v/v) ‘Micropur Forte MF 1000F’ (Katadyn Products, Wallisellen, Switzerland) to prevent microbial growth. *k*_S_ was then calculated as:

(2) $$k_{S}=\left(FxL)/(\mathrm{dP}x{A_{\mathrm{xyl}}}^{-1}\right)$$

where *F* is the maximum flow rate (m^3^ s^−1^), dP is the pressure applied (MPa), *L* is the sample length (m) and *A*_xyl_ is the xylem cross‐sectional area (m^2^).

For vulnerability analyses, samples of *F. sylvatica* were debarked at both ends (~5 cm), while the bark was completely removed from *P. abies* samples to avoid resin clogging of tracheids. Samples were then re‐cut several times under water at both ends until a sample length of 28 cm was reached. The use of a 28‐cm rotor prevented open vessel artefacts in *F. sylvatica* (Choat *et al*., 2010; Torres‐Ruiz *et al*., 2014), but implied that branch samples were 2–3 (*F. sylvatica)* and 3 (*P. abies*) years old. To remove eventual native embolism, samples were attached to the Cori‐Flow system and flushed for 30 min (see above). Samples were then fixed in the 28 cm custom‐built rotor, inside a centrifuge (Sorvall RC‐5; Thermo Fisher Scientific, Waltham, MA, USA) by placing sample ends in transparent plastic reservoirs filled with the same solution as that used for flushing. Cavitron measurements followed the standard method given in Beikircher *et al*. (2010): Hydraulic conductance (*k*) through the sample was measured at successively reduced xylem pressures (P; MPa) induced through a step‐wise increase in the rotational speed. Percentage loss of conductivity (PLC) was then calculated as:

(3) $$\mathrm{PLC}=100\left(1–k_{f}/k_{i}\right)$$

where *k*_i_ is the initial (and therefore maximum) hydraulic conductance (obtained at a P below −0.5 MPa) and *k*_f_ is the hydraulic conductance at the respective P.

Vulnerability analyses were done by plotting PLC *versus* P. Curve fitting and calculation of P inducing 12%, 50% and 80% loss of conductivity (P_12_, P_50_, P_88_), lower and upper confidence intervals, as well as slope of the curve, was performed with the software package ‘fitplc’ in R using the Weibull model (Duursma & Choat, 2017); we fitted one model for each treatment and included replicates as random factor.

### Wood anatomy

Xylem anatomical analyses were made on samples previously used for conductivity or vulnerability analyses (see above). From the centre of three samples per species and treatment, about 2‐cm long pieces were cut and soaked in an ethanol:glycerol:water solution (1:1:1, v/v/v) for a few days. After which cross‐sections (15 μm) were cut using a microtome (Sledge Microtome G.S.L. 1; Schenkung Dapples, Zurich, Switzerland) and stained with safranin‐Astrablue. Tree ring width and anatomical parameters were analysed in the most recent tree rings (*i.e*. 2018 and 2020), on images gained from a digital microscope camera (ProgRes Arktur8; Jenoptik, Jena, Germany) connected to a light microscope (Olympus BX 41, System Microscope; Olympus Austria, Vienna, Austria). Analyses were carried out in transverse sectors (1–2) opposite to the reaction wood using the image analysis software ImageJ (ImageJ 1.45, public domain; National Institutes of Health (NIH), Bethesda, MD, USA). Mean conduit diameters (*d*_mean_, μm) were calculated from 75 to 422 individually measured lumen areas (A) per individual, assuming a circular conduit shape for *F. sylvatica* and a rectangular conduit shape for *P. abies*. Mean hydraulic conduit diameter (*d*_h_) was calculated according to Sperry *et al*. (1994):

(4) $$d_{h}=\frac{\sum d^{5}}{\sum d^{4}}$$

Conduit wall reinforcement ((*t*/*b*)_h_^2^; Hacke *et al*., 2001) was calculated for each transverse section by directly measuring wall thickness (*t*) and conduit diameter (*b*) of the larger conduit within five conduit pairs, averaging *d*_h_ ± 1 μm per sample. To avoid possible biases due to over‐representation of samples with a larger number of conduits, values were first calculated for each sample and then averaged per species, treatment and year.

### Statistical analyses

All datasets and model residuals were tested for normality (Shapiro–Wilk test) and homoscedasticity (Levene test). Intraspecific differences in water potential, sap flow density and ER across all measurement dates for a given treatment, and differences in vulnerability, conductivity and wood anatomical traits between 2018 and 2020, were tested with one‐way ANOVA followed by pairwise Tukey HSD *post‐hoc* test, if significant. Interspecific differences in hydraulic parameters for a given date were tested individually for every date using Student's *t*‐test (equal variances) or Welch *t*‐test (unequal variances) and *P*‐values corrected for family‐wise errors, applying the Bonferroni correction. P_12_, P_50_ and P_88_ values and associated confidence intervals are the outcome of five PLC curves pooled and fitted into a single model per species, treatment and year. Replicates were included as a random factor. All tests were performed at a probability *P* < 0.05 using R version 3.6.1 (R Core Team, 2020). All values presented are given as mean ± SE.

## Results

### Climate and soil volumetric water content

Growing seasons (May–September) of the years under study (2018–2020) showed similar courses in daily mean temperature, between 15 and 18°C. Total precipitation amounted to 448 mm, 389 mm and 452 mm in 2018, 2019 and 2020, respectively. Mean daily VPD was higher in 2018 (6.4 hPa) and 2019 (5.9 hPa), and lower in 2020 (5.0 hPa) (Fig. S1).

During the re‐watering campaign from 25 June to 10 July 2019, mean daily temperatures at canopy height were *ca*. 21 °C, accompanied by a mean VPD of about 10 hPa. Rainfall during the re‐watering period was sporadic and resulted in 20 mm total precipitation (Fig. S1).

**Fig. 2:**
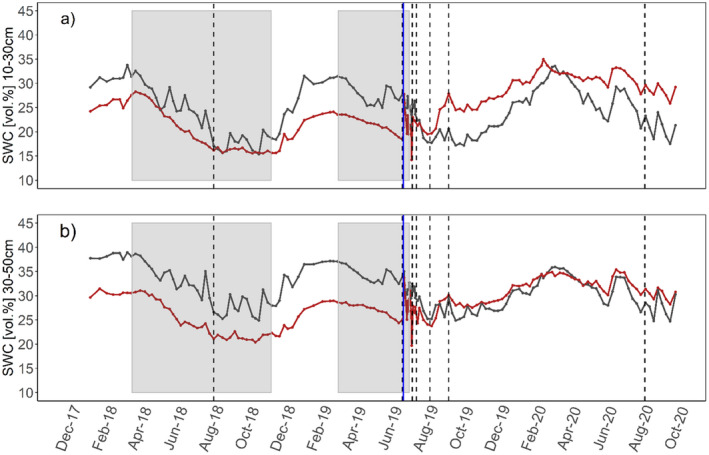
Weekly mean soil water content (SWC vol. %) in 10–30 cm (a) and 30–50 cm (b) depths. SWC is shown for control (black lines) and throughfall exclusion (red lines) plots. Dashed black vertical lines represent date (± 2 days) of ER measurements; solid blue line shows date of re‐watering. Grey‐shaded areas represent periods when roofs were closed. Please note that dates indicated in these graphs are representative for the plots that were re‐watered first, and for the remaining plots these are shifted back for 2 and 4 weeks, respectively (see details on re‐watering in ‘Experimental setup and plant material’).

Until re‐watering in June/July 2019, soil water content (SWC vol. %) at 10–30‐cm depth was about 5–10% lower in TE compared to CO plots. At 30–50‐cm depth, difference in SWC were about 15%, with more pronounced differences during summer months (Fig. 2). Before re‐watering, rainfall resulted in visible SWC peaks in CO plots, but not in TE plots, demonstrating successful throughfall exclusion. After re‐watering, SWC of TE plots quickly recovered in both soil layers, reaching similar (30–50 cm) or even higher (10–30 cm) water content than in CO plots. This trend was still evident in August 2020.

### Pre‐dawn water potential and xylem sap flow density

**Fig. 3:**
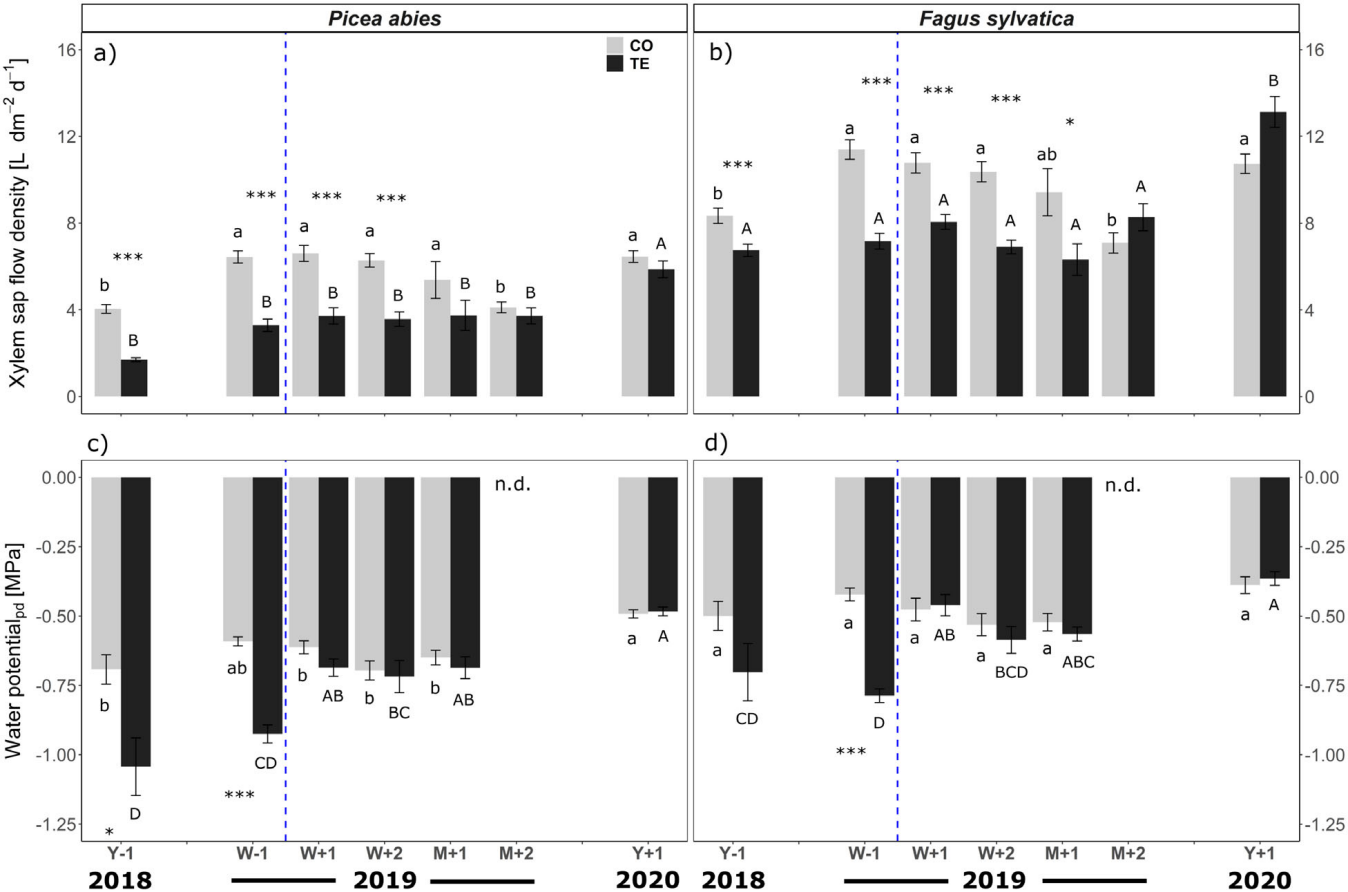
Xylem sap flow density (a, b) and pre‐dawn water potential (c, d) for *Picea abies* (left panels) and *Fagus sylvatica* (right panels) growing on control (grey bars) and throughfall exclusion (black bars) plots, respectively (n = 6–8 trees species^−1^ treatment^−1^). *x*‐axis show measurement periods (Y‐1 = 1 year before and W‐1 = 1 week before re‐watering; W + 1 = 1 week, W + 2 = 2 weeks, M + 1 = 1 month, M + 2 = 2 months and Y + 1 = 1 year after re‐watering). Asterisks indicate statistically significant differences between TE and CO trees on a given date. Significant differences across all dates and within a given treatment of one species are indicated by lowercase (CO) and uppercase (TE) letters, respectively (*P* < 0.05). Blue vertical dashed line indicates re‐watering of plots. No water potential data were available for M + 2. Mean ± SE.

Overall, in *P. abies*, Ψ_pd_ ranged from −0.49 to −0.70 MPa, and − 0.48 to −1.0 MPa in CO and TE trees, respectively (Fig. 3c). At Y‐1 and W‐1, Ψ_pd_ of TE trees was significantly lower (*i.e*. more negative) compared to CO trees. In the following period, similar values were observed for trees of both groups. Mean sap flow density was between 4.0 and 6.6 l dm^−2^ day^−1^ in CO, and between 1.7 and 5.9 l dm^−2^ day^−1^ in TE trees (Fig. 3a). Up to and in W + 2, flux rates were significantly higher in CO than in TE trees, but the differences between treatments diminished thereafter.

In *F. sylvatica*, Ψ_pd_ ranged between −0.39 and − 0.53 and between −0.37 and − 0.79 MPa in CO and TE trees, respectively. Mean sap flow density ranged from 7.1 to 11.4 and from 6.3 to 13.1 l dm^−2^ day^−1^ in CO and TE trees, respectively. Most pronounced differences in sap flow density between CO and TE trees were found 1 week before re‐watering (W‐1; Ψ_pd_), 1 year before (Y‐1) and 1 month after (M + 1) re‐watering (sap flow density).

### Electrical resistivity tomography

**Fig. 4:**
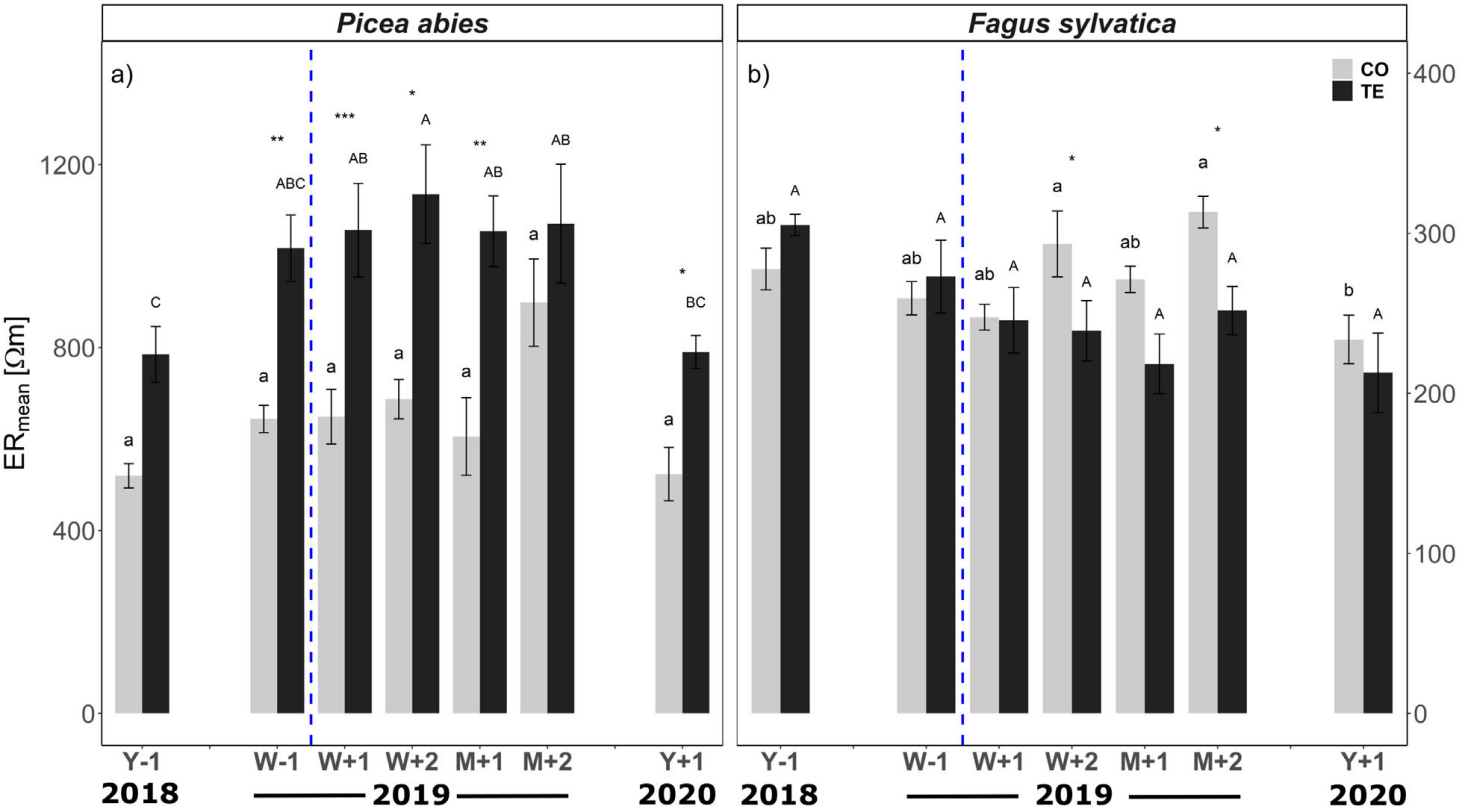
Mean cross‐sectional electrical resistivity (ER_mean_) of P. abies (a) and F. sylvatica (b) trees growing on control (grey bars) and throughfall exclusion (black bars) plots, respectively (n = 6–8 trees species^−1^ treatment^−1^). x‐axis show measurement (Y‐1 = 1 year before and W‐1 = 1 week before re‐watering; W + 1 = 1 week, W + 2 = 2 weeks, M + 1 = 1 month, M + 2 = 2 months and Y + 1 = 1 year after re‐watering). Asterisks indicate statistically significant differences between TE and CO trees on a given date. Significant differences across all dates and within a given treatment of one species are indicated by lowercase (CO) and uppercase (TE) letters, respectively (*P* < 0.05). Blue vertical dashed line indicates re‐watering of plots. Please note individual y‐axis scale for each species. Mean ± SE.

Mean cross‐sectional electrical resistivity (ER_mean_) of *P. abies* control trees was 605 ± 51 Ωm, except in September 2019 (*i.e*. M + 2) when values of 899 ± 95 Ωm were reached (Fig. 4a). At each measurement date, higher ER_mean_ values were found for TE than for CO trees. In both CO and TE trees, similar values were found for Y‐1 and Y + 1, with ER_mean_ of TE trees about 1.5‐fold higher than in CO trees.

In *F. sylvatica*, ER_mean_ was about three‐fold lower than in *P. abies*. Until re‐watering, ER_mean_ tended to be higher in TE trees, but even at maximum drought stress (*i.e*. Y‐1), differences were not statistically significant. In the following period, TE trees showed similar or lower values than CO trees, but the differences were significant only at W + 2 and M + 2. In both treatments, ER_mean_ at Y + 1 was lower than at Y‐1, but an opposite trend was observed (*i.e*. lower ER_mean_ in TE trees).

### Vulnerability to drought‐induced embolism and specific hydraulic conductivity

**Fig. 5:**
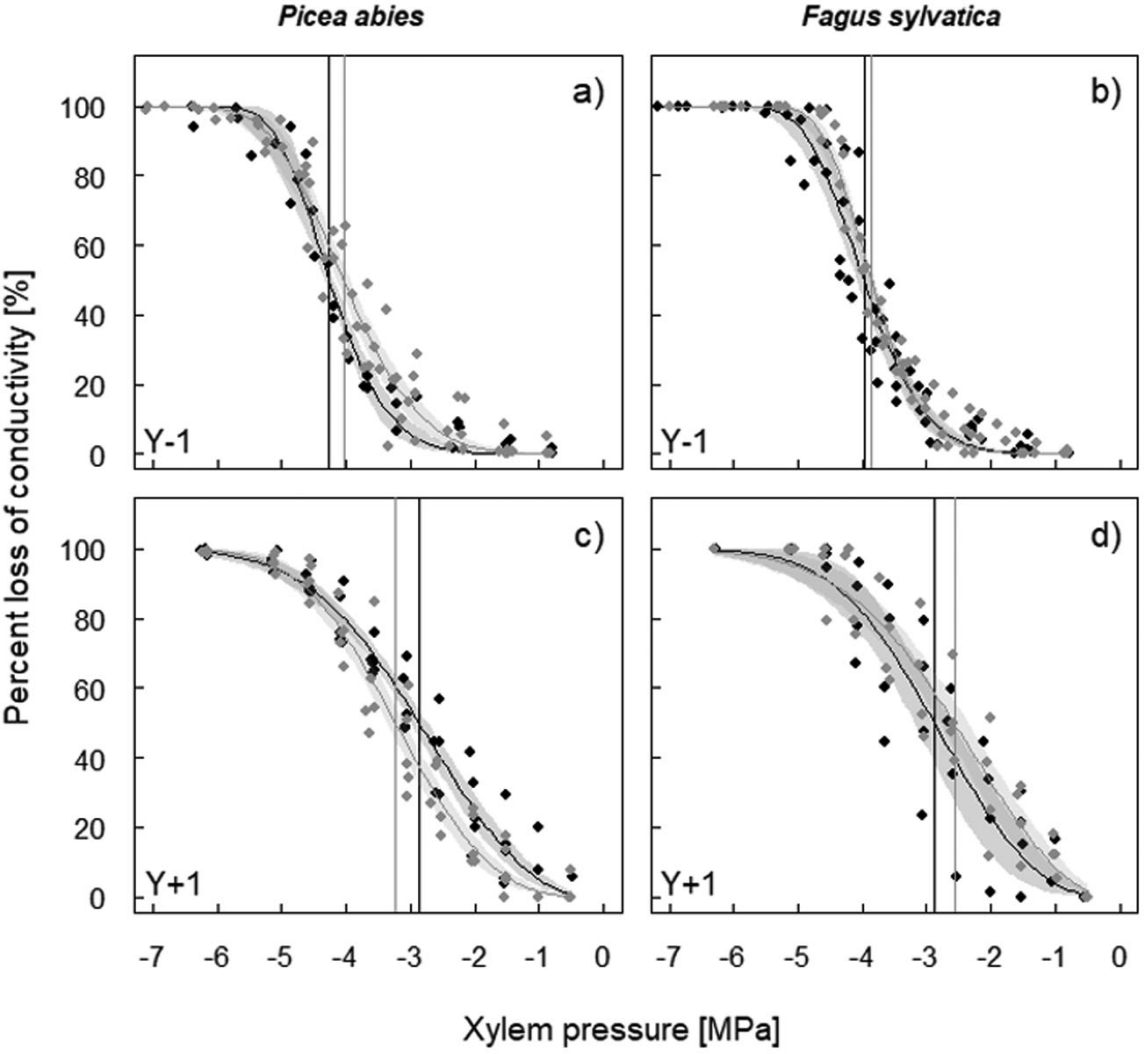
Percentage loss of hydraulic conductivity *versus* xylem pressure of *P. abies* (a, c) and *F. sylvatica* (b, d) trees growing on control (grey) and throughfall exclusion (black) plots 1 year before (Y‐1; a, b) and after (Y + 1; c, d) re‐watering (n = 5 trees species^−1^ treatment^−1^). Vertical lines indicate water potentials inducing 50% loss of conductivity. Shaded areas represent the 95% bootstrapped confidence interval for fitted curves.

At Y‐1, TE trees of *P. abies* tended to be less vulnerable to drought‐induced embolism than CO trees, exhibiting overall lower vulnerability thresholds (P_12_, P_50_, P_88_; Fig. 5a, c, Table 1). At Y + 1, vulnerability thresholds of both CO and TE trees shifted towards significantly less negative water potentials than at Y‐1, with TE trees being slightly more vulnerable than CO trees. Furthermore, slopes of the vulnerability curves were steeper in 2018 (Table 1; no significant differences between treatments). On both dates, Y‐1 and Y + 1, specific hydraulic conductivity (*k*_S_) tended to be lower in TE trees (not significant).

**Table 1:** Hydraulic parameters of branches of *P. abies* and *F. sylvatica* trees growing on control (CO) and throughfall exclusion (TE) plots 1 year before (August 2018; Y‐1) and 1 year after (August 2020; Y + 1) re‐watering (n = 5 trees species^−1^ treatment^−1^). Mean (CI 2.5% /CI 97.5%) for vulnerability thresholds and mean ± SE for specific hydraulic conductivity.

		August 2018 (Y‐1)		August 2020 (Y + 1)	
		CO	TE	CO	TE
*P. abies*	P_12_ (MPa)	−2.90^a^ (−2.70 / −3.10)	−3.24^a^ (−3.12 / −3.53)	−1.97^b^ (−1.73 / −2.10)	−1.48^b^ (−1.25 / −1.64)
	P_50_ (MPa)	−4.01^a^ (−3.94 / −4.15)	−4.22^a^ (−4.18 / −4.34)	−3.23^b^ (−3.09 / −3.36)	−2.86^b^ (−2.73 / −2.99)
	P_88_ (MPa)	−4.99^a^ (−4.86 / −5.25)	−5.04^a^ (−4.80 / −5.28)	−4.52^a^ (−4.38 / −4.79)	−4.46^a^ (−4.31 / −4.71)
	*a*	43.4^a^	57.3^a^	34.3^ab^	29.8^b^
	*k*_S_ (cm^2^ s^−1^ MPa^−1^)	9.8 ± 2.7^a^	7.0 ± 2.0^a^	6.6 ± 0.8^a^	5.7 ± 0.4^a^
*F. sylvatica*	P_12_ (MPa)	−3.03^a^ (−2.91 / −3.15)	−3.14^a^ (−2.88 / −3.22)	−1.78^b^ (−1.02 / −1.87)	−1.71^b^ (−1.21 / −1.97)
	P_50_ (MPa)	−3.85^ab^ (−3.80 / −3.91)	−3.98^a^ (−3.86 / −4.09)	−2.98^ab^ (−2.67 / −3.44)	−2.88^b^ (−2.60 / −3.18)
	P_88_ (MPa)	−4.52^a^ (−4.43 / −4.66)	−4.65^a^ (−4.54 / −4.97)	−4.35^a^ (−4.21 / −6.02)	−4.14^a^ (−3.93 / −4.75)
	*a*	63.2^a^	54.0^a^	29.3^b^	32.7^ab^
	*k*_S_ (cm^2^ s^−1^ MPa^−1^)	34.1 ± 6.7^a^	32.2 ± 4.1^a^	22.6 ± 2.2^a^	32.7 ± 8.5^a^

Letters indicate intraspecific significant differences across treatments and years (P < 0.05).

Xylem pressure inducing 12%, 50% and 88% loss of conductivity (P_12_, P_50_, P_88_) + lower and upper confidence interval (CI 2.5% /CI 97.5%); slope of vulnerability curves (*a*) and specific hydraulic conductivity (*k*_S_).

Moreover, in *F. sylvatica*, lower vulnerability thresholds at Y‐1 and at Y + 1 in TE trees were observed, as well as steeper curves in Y‐1, with even less pronounced differences than in *P. abies* (Fig. 5b, d, Table 1). In August 2018 (Y‐1), *k*_S_ was similar in CO and TE trees, while in 2020 (Y + 1), TE trees tended to have 1.4‐ fold more conductivity than CO trees (not significant).

### Wood anatomy

In CO and TE *P. abies* trees, annual ring width of branches at Y‐1 was about half that at Y + 1 (Table 2). In both years, branches of TE trees tended have reduced growth compared to branches of CO trees. Growth differences were more pronounced in Y‐1 (37% lower annual ring width in TE trees) than in Y + 1 (13% lower annual ring width). There were no differences in mean conduit diameter and mean hydraulic conduit diameter (d_mean_, d_h_), neither between treatments nor between years. In contrast, 1 year after re‐watering, cell wall reinforcement ((t/b)_h_^2^) was about 1.9‐fold higher than during maximum drought stress, with no differences between treatments.

**Table 2:** Branch xylem anatomical parameters for *P. abies* and *F. sylvatica* trees growing on control (CO) and throughfall exclusion (TE) plots 1 year before (August 2018; Y‐1) and 1 year after (August 2020; Y + 1) re‐watering (n = 3 trees species^−1^ treatment^−1^). Mean ± SE.

		August 2018 (Y‐1)		August 2020 (Y + 1)	
		CO	TE	CO	TE
*P. abies*	Ring width (μm)	225 ± 88^a^	143 ± 80^a^	419 ± 27^a^	368 ± 89^a^
	*d*_mean_ (μm)	11.2 ± 0.4^a^	10.8 ± 0.4^a^	10.9 ± 0.2^a^	10.4 ± 0.6^a^
	*d*_h_ (μm)	13.7 ± 0.5^a^	12.8 ± 0.5^a^	13.0 ± 0.3^a^	12.8 ± 0.7^a^
	(t/b)_h_^2^	0.029 ± 0.000^a^	0.026 ± 0.004^a^	0.046 ± 0.000^b^	0.055 ± 0.001^b^
*F. sylvatica*	Ring width (μm)	1136 ± 229^a^	452 ± 194^a^	793 ± 123^a^	879 ± 106^a^
	*d*_mean_ (μm)	23.4 ± 1.2^a^	20.8 ± 1.0^a^	20.4 ± 1.1^a^	21.0 ± 1.4^a^
	*d*_h_ (μm)	31.5 ± 1.1^a^	29.3 ± 0.5^a^	27.5 ± 2.3^a^	25.2 ± 1.8^a^
	(t/b)_h_^2^	0.008 ± 0.001^a^	0.009 ± 0.002^a^	0.018 ± 0.002^b^	0.015 ± 0.002^ab^

Letters indicate intraspecific significant differences across treatments and years (*P* < 0.05).

mean conduit diameter (d_mean_) mean hydraulic diameter (d_h_); conduit wall reinforcement ((t/b)_h_^2^).

In *F. sylvatica*, TE tree increment in branch annual ring width was about twice that at Y + 1 than at Y‐1. However, Y + 1 increment values were lower than at Y‐1 in CO trees (Table 2). At Y‐1, d_mean_ and d_h_ tended to be smaller in TE trees, while at Y + 1, d_mean_ of TE trees was even slightly higher. As found in *P. abies*, in both treatments (t/b)_h_^2^ was significantly higher at Y + 1 than at Y‐1, with no differences between treatments (Table 2).

## Discussion

Here we show that repeated, long‐term summer droughts impact water relations of mature trees, although the extent and the ability to recover is species‐specific. Differences between drought‐stressed and control trees were more pronounced in *P. abies* compared to *F. sylvatica*. One year after re‐watering, the latter trees fully recovered, while *P. abies* showed pronounced legacy effects in trunk water reserves.

### Effects of repeated long‐term summer droughts on tree hydraulics

Five years of artificially induced summer drought resulted in lower pre‐dawn water potentials (Ψ_pd_) and sap flow densities in which differences between CO and TE trees were more pronounced in *P. abies* than in *F. sylvatica*. Compared to CO trees, Ψ_pd_ (though moderate) and sap flow density were 34% and 58% lower in TE trees of *P. abies* and 29% and 19% lower in *F. sylvatica*, respectively (Fig. 3; see Y‐1). Notably, in both species and for both treatments, considerably lower Ψ_pd_ values (< −1.3 MPa) were reported in August 2015, *i.e*. the second year of summer drought stress; while in the third year (August 2016), values were similar to those in 2018 (Tomasella *et al*., 2018). The high interannual differences can be related to climate conditions, with less precipitation and lower soil water content in July 2015 (Grams *et al*., 2021) This clearly demonstrates the dependence of drought impacts on overall (preceding) climate conditions.

Observed differences in Ψ_pd_ and sap flow density also correspond to trunk water content, as analysed with ER tomography. The ER technology proved to be a promising tool for non‐destructive analysis of tree water content and health status (Humplík *et al*., 2016; Bär *et al*., 2019; Ganthaler *et al*., 2019). However, interpretation of tomograms is complex, variation in impact of factors on ER (soil moisture, wood density, electrolytes) hampers direct comparison between species, which has considerable consequences for the monitoring of ER over time. Thus, the interpretation of tomograms must consider the time when the measurements were taken, because wood properties or xylem sap composition and chemistry change seasonally (Bär *et al*., 2019). In our study, tomograms W‐1, W + 1 and W + 2 were measured in spring, Y‐1, M + 1 and Y + 1 in midsummer and M + 2 in autumn, so that observed changes over time might also reflect seasonality. However, the comparison of CO and TE plants on any given date still allows resolution of the impact of throughfall exclusion and subsequent re‐watering. Interpretation of tomograms is further complicated by the fact that slight deviations in tomograms can arise from slight variations in measurement height. Especially in conifers, resin often prevents repeated measurements at the same height. Finally, pathogens can also affect ER patterns (Humplík *et al*., 2016), necessitating multiple replicates (*i.e*. measurements on many similar individuals per species and/or treatment) to enable reliable physiological insights *via* ER tomograms.

In *P. abies*, there was substantially higher cross‐sectional ER (ER_mean_) in TE compared to CO trees (Fig. 4a; Y‐1), indicating drought‐induced reductions in trunk water content. In contrast, no significant differences were observed in *F. sylvatica* (Fig. 4b; Y‐1). Water content is a major factor influencing ER, particularly in conifers. Accordingly, Bär *et al*. (2019) demonstrated that ER patterns in *P. abies* are mainly governed by moisture content, while Ganthaler *et al*. (2019) reported a clear relationship between decreasing water potentials and increased ER in *P. abies* and *F. sylvatica*. Apart from moisture content, ER is also influenced by electrolyte content and wood density, where the impact of each of these components has been found to be species‐specific (Bär *et al*., 2019). Indeed, the ER pattern of *P. abies* in this study differs slightly from patterns found at other sites (*e.g*. Humplík *et al*., 2016; Bär *et al*., 2019; Ganthaler *et al*., 2019), as no highest resistivity values were observed in the centre of stems, but in the centre of the radius. This may be because all the *P. abies* trees at our site were planted at the same time and, as they started growth under optimal conditions, the wood density was lower towards the stem centre but increased as the trees aged and had to compete for light and water resources. In contrast, in *F. sylvatica* the slightly higher ER_mean_ in TE trees could be attributed to the marginally lower Ψ_pd_ but also to the higher wood density, because of marginally lower conduit diameters and higher cell wall reinforcement (Table 2; Jyske *et al*., 2010; Montwé *et al*., 2014).

Surprisingly, hydraulic efficiency and safety, as well as related anatomical parameters, differed only slightly between treatments in both studied species. In August 2018 (Y‐1), the specific hydraulic conductivity (*k*_S_) was only slightly lower in TE compared to CO trees, corresponding to minor differences in conduit diameters (Tables 1, 2). Also, water potentials inducing 50% loss of hydraulic conductivity (P_50_) were only 0.21 MPa and 0.13 MPa lower in TE trees of *P. abies* and *F. sylvatica*, respectively, and differences in cell wall reinforcement were negligible (Tables 1 and 2, Fig. 5). Observed differences were thus less than in August 2016, when values of 0.35 MPa (*P. abies*) and 0.4 MPa (*F. sylvatica*) lower P_50_ in TE trees were observed (Tomasella *et al*., 2018). However, in 2018, the vulnerability threshold were overall lower than in 2016, indicating some interannual variability in hydraulic safety. Overall, the values also indicated a comparatively high drought tolerance of the studied trees. Tree P_50_ values usually range between −3.4 MPa and − 4.6 MPa in *P. abies* (Mayr & Rosner, 2011; Tomasella *et al*., 2018; Rosner *et al*., 2019a; Rosner *et al*., 2019b; Arend *et al*., 2021) and between −2.8 MPa and −3.8 MPa in *F. sylvatica* (Hacke & Sauter, 1995; Cochard *et al*., 1999; Lemoine *et al*., 2002; Herbette *et al*., 2010; Wortemann *et al*., 2011; Hajek *et al*., 2016; Schuldt *et al*., 2016; Stojnic *et al*., 2018; Dietrich *et al*., 2019; Leuschner, 2020; Walthert *et al*., 2021). In the current study, P_50_ at Y‐1 was −4.0 MPa and − 4.2 MPa in *P. abies* CO and TE trees, respectively, and in *F. sylvatica* was −3.9 MPa and − 4.0 MPa, respectively (Table 1). These vulnerability thresholds were obviously sufficient to prevent embolism formation, even under prolonged and repeated artificial drought, considering the observed moderate Ψ_pd_ (Fig. 4). Hence, only about 2% (*P. abies*) and 19% (*F. sylvatica*) loss of conductivity was due to embolism in summer 2018 (data not shown).

Overall, the entire set of hydraulic and anatomical analyses performed indicated only moderate stress intensities after 5 years of artificial summer drought as well as only small hydraulic adjustments in the two study species. It is, however, remarkable that effects in *F. sylvatica* were clearly less pronounced than in *P. abies* trees (even though both species were growing in the same plots), and this may be related to water uptake. First, there is evidence that *F. sylvatica* is able to reduce root water potential under drought conditions, which consequently allows water uptake even in relatively dry soils (Leuschner, 2020). Second, previous studies also found that *F. sylvatica* produces small diameter roots with high conductivity (larger vessel diameter), thus allowing exploitation and efficient use of water resources in desiccating soils (Coners & Leuschner, 2005; Leuzinger *et al*., 2005; Peiffer *et al*., 2014; Leuschner, 2020). Third, as demonstrated by Zwetsloot & Bauerle (2021) at the same study site, *F. sylvatica* showed increased fine root production in deeper soil layers, which probably enabled these trees to reach water reserves that were not accessible to *P. abies*. Accordingly, *P. abies* (as indicated in our ER tomography analyses) buffered increasing drought stress by depletion of internal water reserves. Substantial depletion of internal water reserves of *P. abies* when faced with drought (especially European 2018 drought) has also been reported by Salomón *et al*. (2022). In contrast, water status and trunk water content remained stable in *F. sylvatica*.

### Ability to recover and legacy effects after long‐term summer drought

**Fig. 6:**
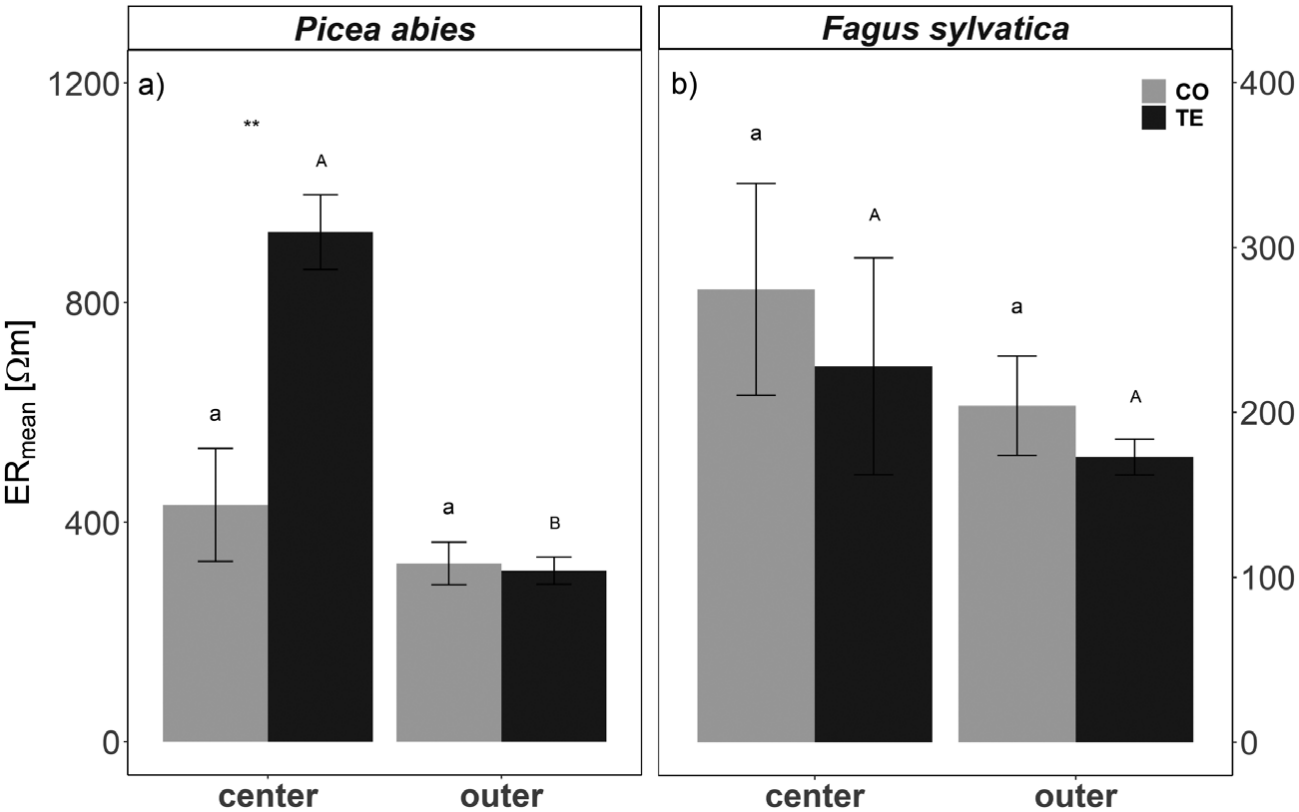
Mean electrical resistivity (ER_mean_; Ohm m) in centre *versus* outer wood of *P. abies* (a) and *F. sylvatica* (b) in 2020 (Y + 1). Centre wood is defined as 0–80% of radial position from trunk centre; outer wood is defined as 80–95% of radial position from trunk centre. Asterisks indicate significant differences between CO (grey bars) and TE (black bars) trees; significant differences across all dates and within a given treatment of one species are indicated by lowercase (CO) and uppercase (TE) letters, respectively (*P* < 0.05). Mean ± SE. Please note individual *y*‐axis scale for the respective species.

In June/July 2019 all plots were re‐watered *via* drip irrigation over 2 weeks (for details see section ‘Experimental Setup’ and Grams *et al*., 2021). Once the very high hydrophobicity of the upper soil layer was overcome, re‐wetting of the deeper clay soil layers was very rapid and homogenous, and, with a higher amount of water added to TE plots, the formerly stressed plots became wetter than the CO plots (Grams *et al*., 2021). Re‐watering led to an almost instant increase in soil volumetric water content (SWC; Fig. 2). Consequently, there was a rapid equilibration in Ψ_pd_ between CO and TE trees, whereas the equilibrium in sap flow density between CO and TE trees was delayed for about 2 weeks in *P. abies* and 1 month in *F. sylvatica* after re‐watering (Fig. 3). In August 2020 (*i.e*. 1 year after re‐watering; Y + 1), most of analysed parameters indicated full recovery of TE trees: Ψ_pd_ was similar in CO and TE trees of both study species (Fig. 3). In *P. abies*, the same was true for sap flow density, while in *F. sylvatica* sap flow density of TE trees even exceeded that of CO trees by 2.4 l dm^−2^ day^−1^. Also, growth parameters indicated complete recovery of both species. While *P. abies* and *F. sylvatica* showed reduced growth under drought in 2018, there were no significant differences in branch ring width in August 2020 (Table 2). Again, TE trees of *F. sylvatica* even tended to grow better than CO trees. We consider that these TE trees profited from stimulated root growth during the drought. Strikingly, both species showed significant shifts in vulnerability thresholds towards less negative water potentials. This was similar in CO and TE trees of both species, again indicating interannual variability (Table 1). Interannual differences in P_50_ were probably based on differences in growth conditions (*e.g*. see SWC of CO plots in Fig. 1) leading to structural changes in the xylem. As conduit diameters were similar in 2018 and 2020, it is likely that the shifts were related to cell wall reinforcement (Table 2) or changes in pit architecture, such as pit pore size, pit membrane thickness, torus overlap and flexibility (Delzon *et al*., 2010; Jansen *et al*., 2012; Losso *et al*., 2018). Neither in 2018 nor 1 year after re‐watering in 2020, were significant differences between TE and CO plants found. However, it should be mentioned that use of 2‐ to 3‐year‐old samples for vulnerability analyses (see Material and Methods) means that possible differences in 2020 might have been underestimated as samples comprised one (*F. sylvatica*) or two (*P. abies*) growth rings that had developed under drought conditions. In contrast to the hydraulic and anatomical parameters mentioned above, which all indicated full recovery, ER tomography revealed relevant legacy effects in *P. abies*: 1 year after re‐watering (Y + 1), ER_mean_ of the TE trees was still lower than that in CO trees (Fig. 4). With respect to the above‐mentioned methodological uncertainties, this was mainly related to ER in the trunk centre (Figs. 6a; Fig. S2). We assume that under drought, water was shifted from internal water reserves to the sapwood, thus buffering water deficits and avoiding development of critical water potentials. This is in line with previously reports, which demonstrated trees can temporarily buffer transpiration losses using internal water reserves (Scholz *et al*., 2011; Hu *et al*., 2018; Manrique‐Alba *et al*., 2018; Mantova *et al*., 2021; Salomón *et al*., 2022). Our findings indicate that *P. abies* was not able to refill the large (and thus) important water reserves in the trunk even 1 year after re‐watering of TE plots. It remains to be studied whether limited radial transport capacity (caused by drought) prevented the restoration of trunk water reserves (Pfautsch *et al*., 2015; Mason *et al*., 2016), or whether the time for restoration was insufficient. The reduced water content in the trunk centre obviously did not affect sap flow density in the sap wood, but the reduced internal reserves might be critical during future drought events. In contrast to *P. abies*, *F. sylvatica* showed rather low resistivities in CO and TE trees 1 year after re‐watering and therefore no depletion of internal water reserves. Notably, in 2020 several tomograms for beech (CO and TE trees) indicated pathogen infestation (decrease in resistivity in the stem centre) due to intensive sampling at the Kroof experimental site for several other studies. However, removing the affected trees from the analysis did not significantly alter the outcomes (similar ER_mean_ as in 2018 and 2019; no significantly lower ER in CO trees). Thus, we expect *P. abies* to be more affected than *F. sylvatica* by repeated drought events, even though some hydraulic measured parameters seemed to fully recover after a drought event. The long‐term depletion of trunk water reserves might be a relevant legacy effect that determines survival in subsequent droughts.

## Conclusion

The throughfall exclusion experiment described here revealed overall moderate but species‐specific hydraulic effects upon repeated long‐term summer drought events. This indicates that mature trees can withstand limitations to water supply during summer over longer periods, and points towards the important role of winter precipitation for maintaining the annual water balance. Re‐watering led to a rapid recovery in many hydraulic parameters, while trunk water content remained low in *P. abies*, which will weaken this tree's potential to withstand future droughts. A better understanding of tree water reserves and capacitance dynamics will be essential to estimate the performance of adult forest trees under global change.

## Supporting information


**Figure S1** Daily mean air temperature at canopy height (a), vapour pressure deficit at canopy height (VPD; b) and daily sum of precipitation (c). Light‐blue shaded area indicates re‐watering period, dotted vertical lines represent measurement periods (Y‐1, W‐1, W + 1, W + 2, M + 1, M + 2, Y + 1). Please note that dates indicated in these graphs are representative for the plots that were re‐watered first, and are shifted back 2 and 4 weeks, respectively for the remaining plots (see details on re‐watering in ‘Experimental setup and plant material’). Break in data from October 2019–January 2020 due to maintenance of devices.

**Figure S2** Example of electrical resistivity tomograms of one individual per species and treatment over time. Areas of high resistivity in tomograms are indicated in red while areas of low resistivity are indicated in blue (see species‐specific scale for electrical resistivity (Ωm)). Please note that displayed resistivity ranges were optimized for visualization and may not represent minimum/maximum ER values.
